# Ni–Doped Pr_0.7_Ba_0.3_MnO_3−δ_ Cathodes for Enhancing Electrolysis of CO_2_ in Solid Oxide Electrolytic Cells

**DOI:** 10.3390/molecules29184492

**Published:** 2024-09-21

**Authors:** Fei Shan, Tao Chen, Lingting Ye, Kui Xie

**Affiliations:** 1 College of Chemistry, Fuzhou University, Fuzhou 350108, China; shanfei@fjirsm.ac.cn (F.S.); chentao@fjirsm.ac.cn (T.C.); 2 Key Laboratory of Design and Assembly of Functional Nanostructures, Fujian Institute of Research on the Structure of Matter, Chinese Academy of Sciences, Fuzhou 350002, China; 3 Fujian College, University of Chinese Academy of Sciences, Fuzhou 350002, China; 4 School of Mechanical Engineering, Shanghai Jiao Tong University, 800 Dongchuan Road, Shanghai 200240, China

**Keywords:** in situ exsolution, CO_2_ electrocatalytic reduction, perovskite electrode, solid oxide electrolysis cells

## Abstract

Solid Oxide Electrolysis Cells (SOECs) can electro-reduce carbon dioxide to carbon monoxide, which not only effectively utilizes greenhouse gases, but also converts excess electrical energy into chemical energy. Perovskite-based oxides with exsolved metal nanoparticles are promising cathode materials for direct electrocatalytic reduction of CO_2_ through SOECs, and have thus received increasing attention. In this work, we doped Pr_0.7_Ba_0.3_MnO_3−δ_ at the B site, and after reduction treatment, metal nanoparticles exsolved and precipitated on the surface of the cathode material, thereby establishing a stable metal–oxide interface structure and significantly improving the electrocatalytic activity of the SOEC cathode materials. Through research, among the Pr_0.7_Ba_0.3_Mn_1−x_Ni_x_O_3−δ_ (PBMN_x_ = 0–1) cathode materials, it has been found that the Pr_0.7_Ba_0.3_Mn_0.9_Ni_0.1_O_3−δ_ (PBMN_0.1_) electrode material exhibits greater catalytic activity, with a CO yield of 5.36 mL min^−1^ cm^−2^ and a Faraday current efficiency of ~99%. After 100 h of long-term testing, the current can still remain stable and there is no significant change in performance. Therefore, the design of this interface has increasing potential for development.

## 1. Introduction

With the development of industrialization and human activities, a great deal of carbon emissions such as carbon dioxide are emitted into the atmosphere, causing a series of climate problems including the greenhouse effect. Among the existing CO_2_ conversion methods, the electrochemical reduction of carbon dioxide has the advantage of possibly being driven by the regulation of power supply voltage and utilization of thermal energy and other energy sources for this process [1,2,3]. Solid Oxide Electrolytic Cells (SOECs) are effective electrochemical devices that can solve the problems of excessive CO_2_ and low utilization of renewable resources [4,5]. Therefore, SOECs not only help to address the greenhouse effect, but also promote the implementation of the dual carbon goals, which is of great significance in addressing the current energy and environmental security situation.

So far, key elements such as electronic conductivity, ionic conductivity, and structural stability of substances can affect the electrolytic performance of a SOEC [6,7,8,9]. According to existing reports, although structural degradation occurs at high external cathode potentials, Ni–ZrO_2_ stabilized with 8% Y_2_O_3_ (Ni–YSZ) still exhibits high electrocatalytic activity in CO_2_ conversion. Under high external potential, carbon deposition occurs during the reduction of CO_2_ to CO, which depends on the influence of the porosity of the electrode [10]. Heidari et al. show that compared with other composite electrodes with different proportions of pure Ba_0.5_Sr_0.5_Co_0.8_Fe_0.2_O_3−δ_ (BSCF), the composite electrode with 20 wt% content of Ce_0.8_Sm_0.2_O_3−δ_ (SDC) showed the highest performance [11]. Yue et al. achieve good results in the electrolysis of pure CO_2_ using La_1−x_Sr_x_Cr_1−y_Mn_y_O_3−δ_ (LSCM). La_0.2_Sr_0.8_TiO_3+δ_ (LST) has certain catalytic ability, good oxygen reduction ability, and high electronic conductivity (conductivity can reach 100 S cm^−1^ at 900 °C), which is also the focus of current research [12,13]. However, its catalytic activity is still lower than that of traditional metal ceramic composite cathodes. Therefore, there is an urgent need to develop perovskite ceramic cathode materials which have excellent stability and catalytic activity.

The chemical formula of perovskite materials is ABO_3_ (The A site is a larger rare earth element (such as Sr^2+^, Ba^2+^, etc.), while the B site is a transition metal element (such as Fe^3+^, Mn^3+^, etc.)), and commonly used cathodes include LSCM, LST, etc. [14]. Due to the perovskite structure, low-valence metal ions can be doped into the A or B sites while generating oxygen vacancies in the perovskite. The presence of oxygen vacancies is crucial for improving the electronic and ionic conductivity as well as catalytic activity of the perovskite [15,16,17]. Therefore, doping at the A or B sites helps to regulate the electrochemical properties of perovskites. Doping vanadium at the La_0.5_Sr_0.5_FeO_3−δ_ (LSF) B site can effectively improve its electrochemical performance. The current density of a La_0.5_Sr_0.5_Fe_1−x_V_x_O_3−δ_-Ce_0.8_Gd_0.2_O_1.9_ (LSFV–GDC) composite cathode reaches 0.6 A cm^−2^ at 1.6 V and 800 °C [18]. RP perovskites have been applied as the anode materials in SOECs mainly because of their mixed ionic and electronic conductivities. The Nd_2_NiO_4+δ_ anode material delivers a current density of 0.64 A cm^−2^ at a cell voltage of 1.3 V at 800 °C, which is three times that of a conventional simple perovskite La_1−x_Sr_x_MnO_3_ (LSM) anode [19].

According to recent research, doping catalysts into the lattice of perovskite materials, followed by nonrepresentational preprocessing such as reduction or polarization, will precipitate catalytically active metal nanoparticles above the lattice surface. By adjusting the stoichiometric ratio of doping, metal nanoparticles can be firmly anchored on the substrate oxide of the cathode material, thereby forming a metal–oxide interface on the electrode surface [20,21,22]. The interface exhibits strong stability in the aggregation and coking resistance of metal nanoparticles [23]. Therefore, it has a certain promoting effect, improving the material’s catalytic capacity. At the same time, perovskite oxide electrodes with in situ exsolution of metal nanoparticles have also received increasing attention [24,25,26]. For example, Ni–doped LSF as a SOEC cathode exhibits good performance and long-term stability during CO_2_ electrolysis. When a voltage of 1.5 V is applied at 850 °C, the current density reaches 1.21 A cm^−2^ [27]. Generally speaking, doping low-valent ions can disrupt the lattice of the original atoms, effectively regulate the concentration of oxygen vacancies, and thereby promote the activation ability of molecules [28].

Conventional SOECs use Ni–YSZ and LSM–YSZ as electrodes. These electrodes, however, suffer from redox instability and coarsening of the Ni electrode along with delamination of the LSM electrode during steam electrolysis. In this work, we used the perovskite material Pr_0.7_Ba_0.3_MnO_3−δ_ (PBM) as the substrate due to its high electronic conductivity, redox stability, and excellent structural properties. Then, we doped Ni at its B site to obtain a sequence of cathode materials. By conducting heat treatment at 750 °C in a reducing atmosphere, in situ exsolution of Ni nanoparticles could be achieved. The metal–oxide interface constructed between the two would generate an extremely stable interface effect, thereby increasing the number of surface responder loci and promoting CO_2_ reduction reactions. Under operating conditions of 850 °C, we used YSZ as an electrolyte and directly measured CO_2_ electrolysis to evaluate the electrocatalytic performance of a Pr_0.7_Ba_0.3_Mn_1−x_Ni_x_O_3−δ_ (PBMN_x_) material. In addition, stability testing of long-term performance was also conducted using symmetric batteries loaded with PBMN_x_.

**Figure 1:**
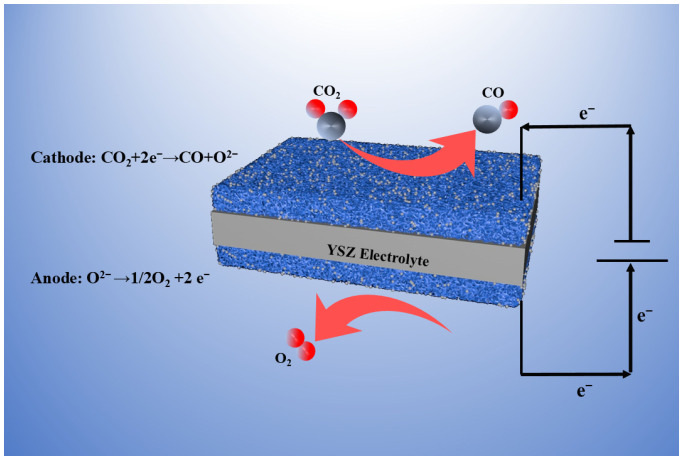
Schematic diagram of system for CO_2_ electrolysis through the SOEC.

Figure 1 shows a complete schematic diagram of the catalytic electrolysis of CO_2_ using the SOEC electrolysis system. From the image, we can see that after a series of reduction treatments, a given mass of metal nanoparticles will precipitate on the electrode materials’ boundary at both ends, thereby constructing a metal–oxide interface and promoting the reaction. We applied specific voltages (1.2 V, 1.4 V and 1.6 V) in a high-temperature environment, and CO_2_ reacts with it in the cathode (feed area) to produce O^2−^ and CO. The generated O^2−^ ions are shifted to the anode boundary (air side) and traverse the tight YSZ electrolyte layer in the middle to generate another reaction, producing O_2_. Different reactions occur on both sides of the battery, with CO_2_ + 2e^−^ → CO + O^2−^ at the cathode end and O^2−^ → 1/2O_2_ + 2e^−^ at the anode end. This reaction not only benefits the achievement of carbon neutrality goals, but also generates a certain amount of available energy for use.

## 2. Results and Discussion

**Figure 2:**
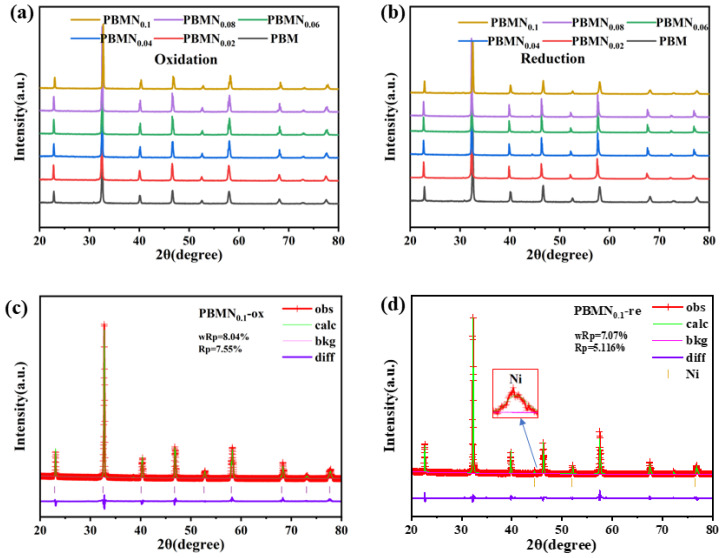
XRD sample spectrum of cathode materials (**a**) oxidized in air and (**b**) reduced in 5% H_2_/Ar; XRD Rietveld refinement patterns of the (**c**) oxidized and (**d**) reduced PBMN_0.1_.

As shown in Figure 2, we analyzed the XRD patterns of the oxidized state obtained by calcination at 1300 °C for 6 h and the reduced state obtained by reduction under 750 °C atmosphere in order to distinguish the crystal structure and in situ exsolution behavior of the Pr_0.7_Ba_0.3_Mn_1−x_Ni_x_O_3−δ_ material. Compared with PDF#98041, the diffraction peak positions of the sintered material in Figure 2a are basically consistent with the peak positions on the PDF card, and there is almost no shift, indicating the synthesis of pure-phase PBM and PBMN. Figure 2b shows the XRD diffraction peaks of PBMN_x_ powder after 20 h of high-temperature reduction at 750 °C using 5% H_2_/Ar gas. By observing this, we can see that the overall peak position has not changed, but a new peak position has emerged around 45°, indicating that the phase transition is reversible. Figure 2c,d indicates that XRD Rietveld refinement is performed on the cathode powder samples of both oxidation and reduction modes using GSAS-II software (gsas2full 4776 (Python 3.7.1 64-bit)). We can see in Figure 2d that a new peak appears around 44.496° by zooming in around the 45° range. After comparison with PDF#87-0712, it is confirmed to be Ni nanoparticles, indicating that Ni nanoparticles have been anchored on the electrode surface, forming a stable metal–oxide interface. In Figures S1 and S2, analysis shows that with the change in doping element ratio, the structure of the crystal itself does not change.

**Figure 3:**
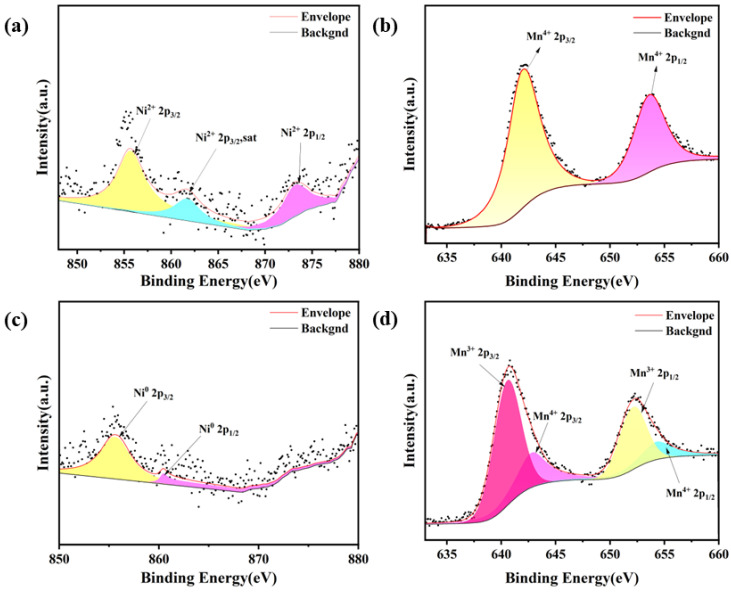
XPS of (**a**) Ni 2p orbitals and (**b**) Mn 2p orbitals in the oxidized state of PBMN_0.1_ material; XPS of (**c**) Ni 2p orbitals and (**d**) Mn 2p orbitals in the reduced state of PBMN_0.1_ material.

The oxidation state of calcined PBMN_x_ powder obtained through liquid-phase combustion and the fluctuations in the valence state values of compounds of Mn and Ni after pretreatment with 5% H_2_/Ar at 750 °C were analyzed by XPS spectroscopy. As shown in Figure 3a,b, oxidized doped Ni exists on the substrate of PBM in the form of Ni^2+^, while Mn exists in the valence state of Mn^+4^. In our analysis, the appearance of two main peaks corresponding to 2p_3/2_ and 2p_1/2_ of Ni^2+^, as well as a satellite peak, is very significant. At positions around 855.8 and 873.5 eV, we can clearly see the peaks of Ni 2p_3/2_ and Ni 2p_1/2_ of Ni^2+^. On the contrary, through the analysis of Figure 3c,d, after 5% H_2_/Ar pretreatment, we find new peaks attributed to Ni 2p_3/2_ and Ni 2p_1/2_ of Ni^0^, respectively [29]. In addition, we have noticed the presence of Mn, which appears in the valence states of Mn^+4^ and Mn^3+^. It is worth noting that in Figures S5 and S6 the binding energy of these two valence states decreases after reduction, which theoretically suggests that reduction treatment can cause a change in valence states, which may lead to lattice distortion and promote ion transport [30]. These findings are of great significance for a deeper understanding of the behavior of materials.

**Figure 4:**
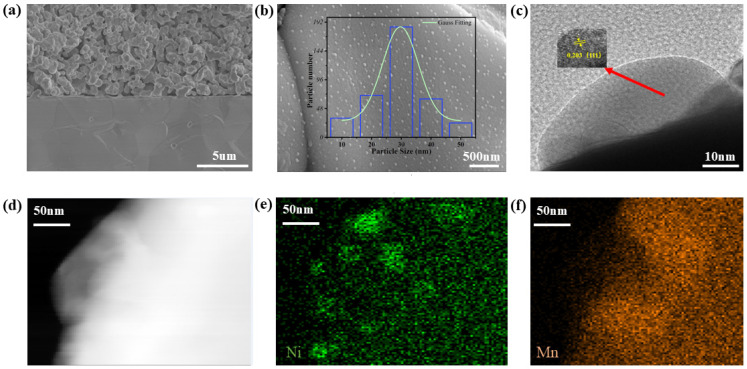
(**a**) SEM images of PBMN_x_-SDC cathode material before electrolysis experiment; (**b**) SEM images of reduced samples with particle size distribution of PBMN_0.1_; TEM image (**c**) of Ni nanoparticles precipitated from exsolution; (**d**) PBMN_0.1_ sample after reduction treatment; (**e**) TEM mapping images of Ni and (**f**) Mn elements.

Figure 4a presents a scanning electron microscope image of the entire cross-section of a single battery. We choose dense YSZ as the electrolyte because it provides excellent oxygen ion conductivity, enabling effective conduction of oxygen ions, and exhibits good stability at high temperatures. In high-temperature environments, the evaporation of organic matter creates the porous characteristics of the electrode, which not only accelerates the diffusion of reaction gases, but also ensures sufficient contact possibility between CO_2_ and the catalyst. Figure 4b shows that after undergoing 5% H_2_/Ar gas reduction, the exsolution phenomenon of Ni nanoparticles in PBMN_0.1_ can be clearly seen, and the particle size is mostly around 30 nm. At the same time, the formed exsolution interface will improve its thermal stability and enhance its electrode activity to a certain extent. Figure 4c shows the TEM graphics of the exsolved nanoparticles in the sample after reduction. After measurement, the lattice spacing of the nanoparticles is 0.203 nm, corresponding to the (111) surface of Ni nanoparticles. Figure 4d–f, Figures S3 and S4 show the energy spectrum analysis and surface scanning performed on the PBMN_0.1_ sample at this magnification. The above studies indicate that Ni has a good desorption effect in oxide surface.

**Figure 5:**
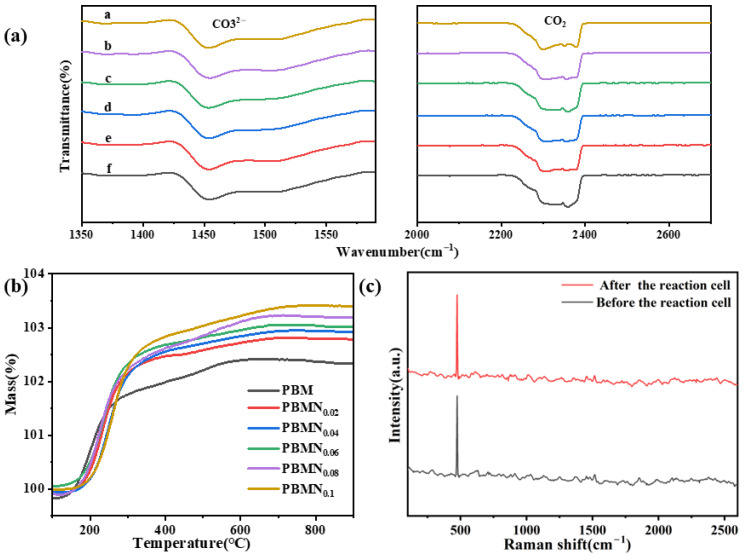
(**a**) In situ FT-IR spectroscopy of CO_2_ using PBMN_x_ cathode material (a: PBMN_0.1_; b: PBMN_0.08_; c: PBMN_0.06_; d: PBMN_0.04_; e: PBMN_0.02_; f: PBM); (**b**) TGA analysis of PBMN_x_ samples; (**c**) For Raman spectra before and after the reaction.

As shown in Figure 5a, all components of this material exhibit CO_2_ peaks around 2200–2400 cm^−1^, indicating that the material has good adsorption capacity for CO_2_. At the same time, a CO_3_^2−^ peak is also observed around 1450 cm^−1^, which is due to the intermediate reaction of CO_2_ adsorbed on the electrode surface. On this basis, oxygen defects are utilized to effectively activate the CO_2_ adsorbed on it, thereby increasing the reaction rate. Meanwhile, utilizing oxygen defects increased oxygen transport capacity and improved the electrochemical activity of SOEC. As shown in Figure 5b, various reduced components are subjected to TGA testing in an air atmosphere. The results indicate that the metal nanoparticles present on the surface of the material electrode have been exsolved, but due to their susceptibility to oxidation, the total mass at high temperature increases with increasing temperature. At low temperature, the sample exhibits varying degrees of weight loss, which was generally attributed to the loss of water, CO_2_, or other impurities adsorbed by the sample. As shown in Figure 5c and Figure S7, we test the Raman spectra of the PBMN_0.1_ cathode material before and after the reaction. After testing and analysis, it can be concluded that after long-term testing, there is no significant change in the Raman spectra, and there is no carbon peak; the experimental results show that this cathode material has good resistance to carbon deposition [31].

**Figure 6:**
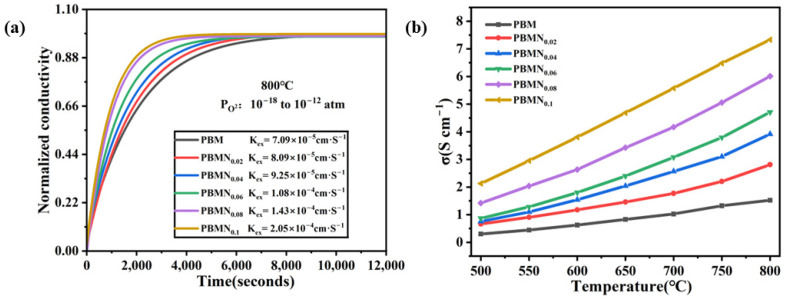
(**a**) Conductivity relaxation curve of bulk samples at 800 °C; (**b**) oxygen ion conductivity of different samples from 500 to 800 °C.

The surface exchange coefficient (reaction rate constant) (K_ex_) is measured by the conductivity relaxation method (ECR). Meanwhile, by analyzing the interface exchange performance of the material, the influence of interface reactions on the direct CO_2_ electrolysis process is explored. This technology has been widely applied to determine the reaction kinetics on electrode materials of solid oxide batteries. The samples required for ECR testing are usually bar shaped blocks, so it can accurately analyze the surface dynamics of CO_2_ direct electrolysis reactions in different cathode materials. The test in Figure 6a is conducted in a reducing atmosphere of 5% H_2_/Ar. When the temperature reaches 800 °C, the atmospheric pressure increases from 10^−18^ atm to 10^−12^ atm. At this time, the K_ex_ value of the PBMN_0.1_ cathode material is measured as 2.05 × 10^−4^ cm s^−1^, which is higher than the K_ex_ value of other cathode materials measured. Through analysis, it can be concluded that the increase in K_ex_ value has a certain impact on the reduction in rebalancing time. The increase in K_ex_ value is caused by the precipitation of anchored nickel metal nanoparticles on the lattice interface surface. The oxygen ion conductivity of different cathode materials is shown in Figure 6b. The oxygen ion conductivity of PBMN_0.1_ is 6–7 times higher than that of PBM. Research has shown that interface reactions in this system play an important regulatory role in oxygen diffusion behavior, and on this basis, metal particles in the exsolved material can also enhance its conductivity, thereby improving its catalytic activity.

**Figure 7:**
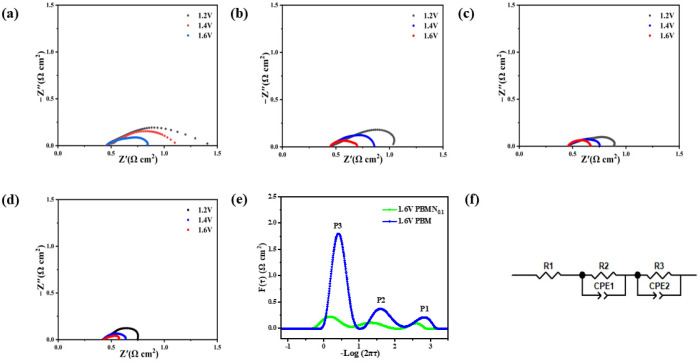
AC impedance based on (**a**) PBM, (**b**) PBMN_0.04_, (**c**) PBMN_0.08_, (**d**) PBMN_0.1_ of electrolyzed CO_2_ at 1.2–1.6 V and 850 °C; (**e**) DRT analysis of impedance spectrum at 1.6V voltage; (**f**) corresponding equivalent circuit diagram.

In order to investigate the resistance characteristics of this material, we conduct AC impedance spectroscopy tests at a temperature of 850 °C and apply voltages of 1.2 V, 1.4 V, and 1.6 V. The values at the intersection of the high-frequency region and the horizontal coordinate correspond to the ohmic impedance Rs. Since the preparation process of different battery monomers is the same, the battery size and electrolyte thickness are approximately equal, and the test environment is consistent, so Rs is approximately equal. The difference between the value of the low-frequency region and the intersection of the transverse axis and the value of Rs is the polarization impedance Rp, which is determined by the electrochemical polarization impedance caused by charge transfer and the concentration polarization impedance caused by diffusion [32]. According to the analysis of the results in Figure 7a–d, the ohmic resistance of various components of this material is almost the same, ranging from 0.43 to 0.45 Ω cm^−2^. This is because a YSZ electrolyte is also used and the thickness is the same. In addition, as the voltage increases in an orderly manner, the polarization resistance of this material correspondingly decreases, indicating that an increase in applied voltage promotes electrode polarization. By calculating its polarization resistance, the polarization resistance of the PBMN_x_ cathode material precipitated by Ni nanoparticles has significantly decreased. As shown in Figure 7d, it can be seen that under the condition of 1.6 V, the polarization resistance of the PBMN_0.1_ (0.132 Ω cm^−2^) cathode material decreases by about 86% compared to the PBM (0.958 Ω cm^−2^) cathode material, showing better performance. In Figure 7e, Distribution of Relaxation Time (DRT) is used to analyze the electrode reaction process. The P1 process occurring in the high-frequency band represents the process of O^2−^ transmission through the YSZ electrolyte, and by comparison it can be seen that the area of the region is roughly the same; this is because the same YSZ electrolyte is used. The P2 process in the middle-frequency band is related to the charge transfer process in the cathode reaction, while the P3 process in the low-frequency band is the adsorption, dissociation, and diffusion process of polarized active CO_2_ on the cathode surface [33]. After doping Ni, the peak area of P2 and P3 processes decreased to different degrees, indicating that the adsorption process and electrochemical reduction process of CO_2_ were improved. Compared with the equivalent circuit diagram in Figure 7f, it can be seen that the electric reduction process of CO_2_ at the cathode is controlled by charge transfer and ion diffusion [34].

**Figure 8:**
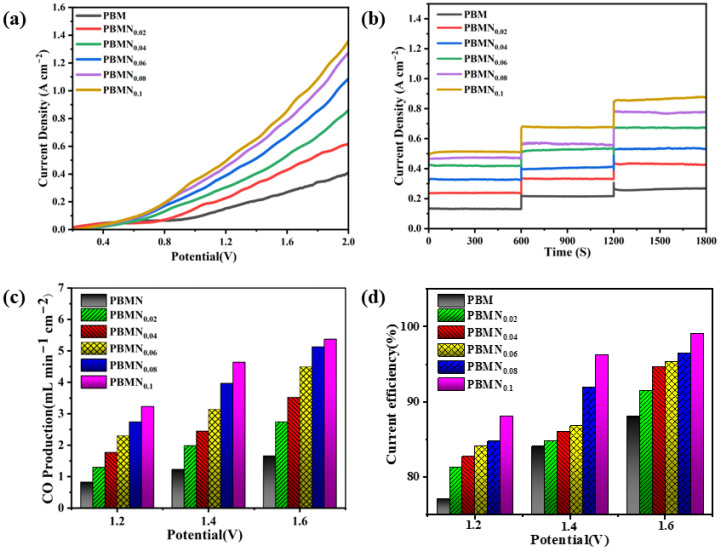
Performance of PBMN_x_-based SOEC at 850 °C and with CO_2_ gas; (**a**) instantaneous voltage current; (**b**) short-term stability testing of current density; (**c**) the productivity of CO; (**d**) the Faraday current efficiency of CO_2_ electrolysis.

Figure 8 shows the analysis of the current and catalytic performance of the six cathode materials through specific experimental steps. Figure 8a shows the instantaneous current density of cathode materials with different components under an applied voltage of 0.2 V to 2.0 V. From the graph, we can see that when the applied voltage reaches 0.6 V, the trend of the curve changes significantly, indicating that the initial voltage for CO_2_ electrolysis of this material is about 0.6 V. At the same time, it can also be seen that as the applied voltage continues to increase, its current density also continues to rise. The current density of the PBMN_0.1_ cathode material increases the most, with a current density of 0.85 A cm^−2^ at 1.6 V. As shown in Figure 8b, this chart meticulously depicts the short-term current performance of different PBMN_x_ materials under voltage environments of 1.2 V, 1.4 V, and 1.6 V. During this brief testing period, the current density remains stable as a rock, which undoubtedly demonstrates the excellent electrochemical stability of single cells. This result not only provides strong data support for the performance evaluation of batteries, but also indicates their potential endurance and reliability in future applications. We can see that the current density of the PBMN_0.1_-SDC cathode material increased by about 3.27 times compared to the current density of the substrate material PBM under an applied voltage of 1.6 V. This is because the Ni nanoparticles that are exsolved and precipitated during the process are firmly fixed on the surface of the PBM cathode, and the concentration of oxygen vacancies increases, resulting in an increase in current density as well. As shown in Figure 8c, we can observe the CO generation rate of different cathode materials under different applied voltages. At 850 °C and an applied voltage of 1.6 V, the CO generation rate of PBMN_0.1_ electrode material reaches 5.36 mL min^−1^ cm^−2^, which is 3.35 times higher than the performance of the PBM base material under the same conditions. Figure 8d illustrates the Faraday current efficiency of different electrode materials under applied voltages of 1.2 V, 1.4 V, and 1.6 V at a temperature of 850 °C. It can be seen that the Faraday current efficiency of the PBMN_0.1_-SDC electrode material can reach ~99% at 1.6 V, which is about 11% higher than that of PBM. This indicates that the interface composed of Ni nanoparticles formed by desorption precipitation does have a certain effect on the improvement of cathode material activity. Meanwhile, as shown in Figure S8, the PBMN_0.1_ cathode material shows the most excellent performance.

**Figure 9:**
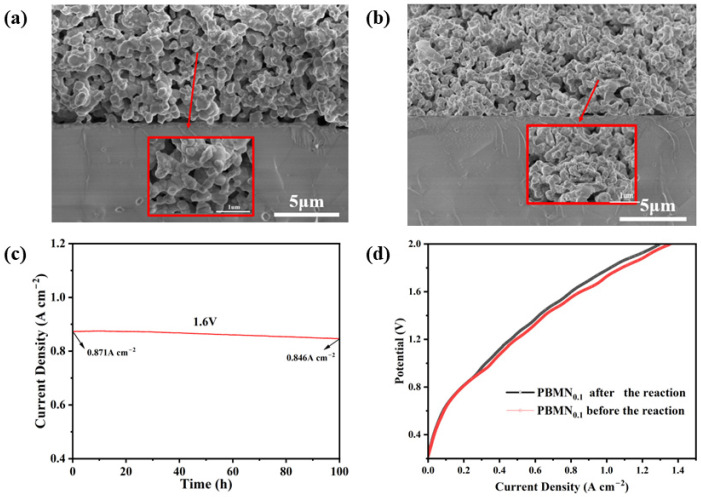
(**a**) SEM image of PBMN_0.1_ cathode material before electrolysis experiment; (**b**) SEM image of PBMN_0.1_ cathode material after electrolysis experiment; (**c**) long-term current testing of SOEC based on PBMN_0.1_ material at 1.6 V; (**d**) polarization curve before and after electrolysis experiment.

As shown in Figure 9a,b, we observed the internal structure of the PBMN_0.1_-SDC electrode material after long-term testing. From the image, we can see that the material can still maintain a stable porous structure after long-term testing and there is no sintering. By zooming in on the image, we can also clearly see that Ni nanoparticles are desorbed from the electrode surface, indicating that they have a certain degree of stability. The interface between the electrode and electrolyte is clear and stable, indicating the stability of the SOEC is good. To further demonstrate the electrochemical stability of the battery, as shown in Figure 9c, we test the long-term performance of PBMN_0.1_ cathode material under an applied voltage of 850 °C and 1.6 V for 100 h. After 100 h of testing, it remains at around 0.846 A cm^−2^, and although there is a slight decrease, the current density of the battery still remains relatively stable. CO_2_ adsorption on perovskite oxides produces various surface carbonate species, and even CO_2_ reacts with perovskite oxides to produce BaCO_3_, which can lead to degradation of perovskite cathodes [35]. The degradation of the cell also depends greatly on the current density of the electrolysis [36]. So, we also analyze the IV polarization curves of the battery before and after the electrolysis experiment, and the polarization curves are almost identical before and after the electrolysis experiment, which also shows that the battery have excellent long-term stability.

## 3. Experiment

### 3.1. Material Preparation

We first prepared Pr_0.7_Ba_0.3_Mn_1−x_Ni_x_O_3−δ_ (x = 0, 0.02, 0.04, 0.06, 0.08, 0.1) cathode material powder using the traditional solid-state synthesis method for basic characterization testing. We mixed Pr_6_O_11_ (Aladdin, Shanghai, China), BaCO_3_ (Aladdin), and MnO_2_ (Macklin, Shanghai, China) in their respective ratios, then added small and numerous steel balls and a certain amount of anhydrous ethanol to the cylinder for shaking. Then, we placed them in a ball mill for treatment. After about 3 h, we removed them to ensure sufficient mixing and poured the liquid into an evaporating dish and placed them in an oven. After drying treatment for 5 h at 60 °C, the powder material was turned into powder. The obtained material would also be subjected to 6 h of heat treatment at 1300 °C.

Secondly, the perovskite material PBMN_x_ powder was compounded using the glycine liquid-phase combustion method for testing. On the basis of different chemical dosage ratios, PrN_3_O_9_·6H_2_O (Aladdin), Ba(NO_3_)_2_ (Aladdin), Mn(NO_3_)_2_·4H_2_O (Aladdin), and Ni(NO_3_)_2_·6H_2_O (SCR, Shanghai, China) were added to a beaker for heating and a rotor was added to it on a magnetic stirrer to continuously mix the substances evenly. Then, during the mixing of the substances, nitric acid was introduced to regulate the pH value to about 1–1.5 to promote exsolution of substances, followed by ammonia to adjust, before finally adjusting the pH to about 2.5–3. After a long wait, a black brown powder was obtained, and then the obtained powder was placed in a crucible and placed in a muffle furnace for 6 h of heat treatment at a high temperature of 1300 °C. Here, we also prepared Ce_0.8_Sm_0.2_O_3−δ_ (SDC) material using the liquid-phase synthesis method. The acquired material was also placed in a muffle furnace and subjected to 4 h of heat treatment at 810 °C.

In order to study the surface dynamics of the cathode material sample PBMN_x_ obtained through EPR technology, we first put the powder material into a mortar and ground it evenly. After weighing a certain amount of sample, the pressure was adjusted to about 12 MPa and held for 30 s according to the grinding tool, and finally, it was pressed into a regular rectangular rod. Next, the compressed sample was heat-treated at 1300 °C for 10 h to obtain dense PBMN_x_ rods with an effective size of approximately 12 (L) × 5 (W) × 2 (H) mm^3^.

Next, we began the preparation of the battery. Firstly, 0.975 g of Pr_0.7_Ba_0.3_Mn_1−x_Ni_x_O_3−δ_ powder and 0.525 g of SDC powder was weighed and placed in a mortar for grinding with a grinding rod until they were completely mixed into a fine powder. Then, 0.3 g of ethyl cellulose and 0.2 g of cassava starch was determined and conflated regularly. Finally, we gradually added translucent pine oil alcohol to the electrode slurry and ground it evenly to obtain an almost viscous electrode slurry. Then, YSZ dust was held down into flake with a semidiameter of approximately 1 cm, and maintained at 1450 °C for 10 h in a muffle furnace to obtain a relatively dense YSZ electrolyte. The battery supported by the obtained YSZ electrolyte would be used for electrochemical testing. The thickness of the YSZ electrolyte sheet was about 4 mm. Then, we evenly mixed electrode slurry points that would be applied on both sides of the YSZ electrolyte sheet. The thickness of the electrode slurry on both sides was about 1 mm. Then, a complete battery was obtained by keeping it at high temperature of 1150 °C for 2 h. Finally, we applied silver paste evenly on both sides of the battery as a collector layer, and placed it in a muffle furnace to calcine at 560 °C for 0.6 h to obtain batteries.

### 3.2. Testing and Characterization

X-ray diffraction (XRD, Rigaku, Miniflex 600, Akishima, Japan) was used to characterize the crystal structure of the synthesized powder (oxidation and reduction states of PBMN_x_). The internal morphology of the oxidized and reduced samples was observed using scanning electron microscopy (SEM, SU8010 HHTNT-536-9424, Hitachi, Tokyo, Japan). A thermal gravimetric analyzer (STA 449 F5, NETZSCH, Selb, Germany) was used to analyze the variation of sample quality with temperature in air. In addition, Raman testing (LabRAM HR Evolution, Horiba, Kyoto, Tokyo) was used to analyze the constancy and anti-coking properties of the substance itself.

The four-probe method was used for electrochemical testing of the battery, mainly including the volt ampere characteristic curve, AC impedance spectrum, and long-term stability testing. The testing instrument used to record the electrochemical properties tested was a Zahner IM6 electrochemical workstation. Firstly, we sealed the battery in a vertical tube furnace to ensure good airtightness and then heated it up to a working temperature of 850 °C. During the testing process, the cathode was in a pure CO_2_ atmosphere with a velocity of flow of 50 mL^−1^ min^−1^, and the anode was exposed to atmosphere. Finally, by connecting the pipeline to the Shimadzu gas chromatograph (Shimadzu, GC2014, Kyoto, Japan), the CO_2_ adsorption capacity and CO concentration generated during the reaction process were analyzed.

## 4. Conclusions

In summary, we doped the B site of the perovskite material and used it as a SOEC cathode for CO_2_ electrolysis. Compared with PBM, its electrochemical performance is significantly improved. The polarization impedance of PBM batteries under 1.6V voltage applied at 850 °C is about 0.96 Ω cm^−2^. After doping with Ni, it decreases to 0.15 Ω cm^−2^, a decrease of about 80%, indicating a significant improvement in the electrolytic performance of PBM batteries after Ni doping. On this basis, nickel metal particles are desorbed on the surface of electrode materials and combined with metal oxides in the matrix to achieve efficient absorption and degradation of CO_2_. The best performing electrode material in this experiment is PBMN_0.1_, with a CO production of 5.36 mL min^−1^ cm^−2^. After 100 h of high-temperature testing, its characteristics remain stable. From this, it can be seen that the exsolution and precipitation of Ni can not only improve the electrochemical performance of electrode materials, but also help maintain the long-term stability of batteries, indicating that PBMN is an efficient and stable cathode material for SOEC CO_2_ electrolysis.

## Data Availability

Data are contained within the article and Supplementary Materials.

## Supplementary Materials

The following supporting information can be downloaded at: https://www.mdpi.com/article/10.3390/molecules29184492/s1, Figures S1 and S2 correspond to the refined diagrams of the oxidation and reduction states of the six cathode materials respectively, and Figures S3–S6 further explain the element content and distribution of the material in general. Figure S7 shows the Raman spectrogram analysis of the material after the reaction, which can show that there is no carbon deposition. Figure S8 gives an overall analysis of the properties of the material, from which it can be seen more intuitively that PBMN_0.1_ shows better performance.
