# Supplementary material for: Ni–Doped Pr0.7Ba0.3MnO3−δ Cathodes for Enhancing Electrolysis of CO2 in Solid Oxide Electrolytic Cells
Source: Molecules. 2024 Sep 21;29(18):4492. doi: 10.3390/molecules29184492 (PMC11435114; doi:10.3390/molecules29184492)
Supplement: Supplementary file 1 [file molecules-29-04492-s001.zip › molecules-3182608-supplementary.pdf]

Supplementary Materials

Article

Ni–Doped $Pr_{0.7}Ba_{0.3}MnO_{3-\delta}$ Cathodes for Enhancing Electrolysis of CO_2 in Solid Oxide Electrolytic Cells

Fei Shan [1,2,3], Tao Chen [1,2,3], Lingting Ye [1,2,3,*] and Kui Xie [1,2,4,*]

[1] College of Chemistry, Fuzhou University, Fuzhou 350108, China; shanfei@fjirsm.ac.cn (F.S.); chentao@fjirsm.ac.cn (T.C.)
[2] Key Laboratory of Design and Assembly of Functional Nanostructures, Fujian Institute of Research on the Structure of Matter, Chinese Academy of Sciences, Fuzhou 350002, China
[3] Fujian College, University of Chinese Academy of Sciences, Fuzhou, 350002, China
[4] School of Mechanical Engineering, Shanghai Jiao Tong University, 800 Dongchuan Road, Shanghai 200240, China
* Correspondence: ltye@fjirsm.ac.cn (L.Y.); xiekui@sjtu.edu.cn (K.X.); Tel.: +86-0591-63179183 (L.Y.)

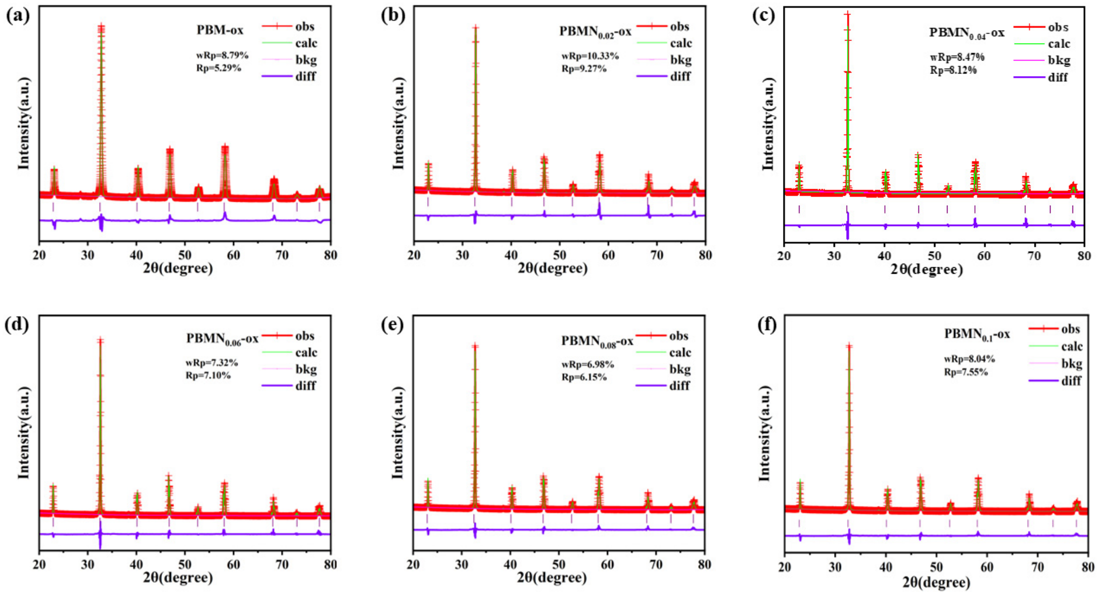

Figure S1. XRD Rietveld refinement patterns of the oxidized in air (a: PBM; b: PBMN$_{0.02}$; c: PBMN$_{0.04}$; d: PBMN$_{0.06}$; e: PBMN$_{0.08}$; f: PBMN$_{0.1}$).

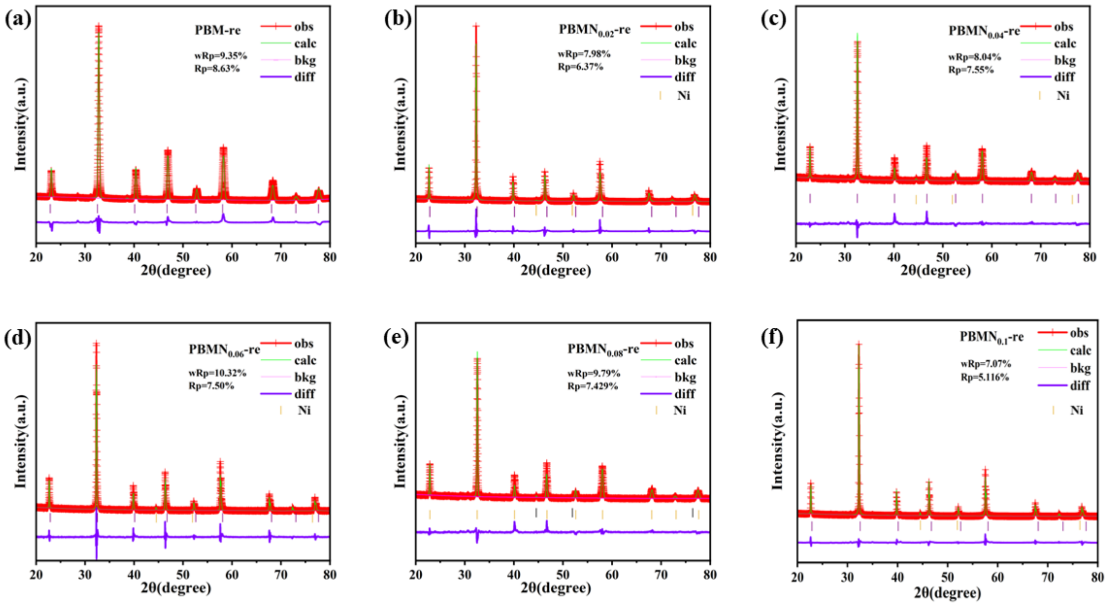

Figure S2. XRD Rietveld refinement patterns of the reduced in 5% H_2/Ar (a: PBM; b: $PBMN_{0.02}$; c: $PBMN_{0.04}$; d: $PBMN_{0.06}$; e: $PBMN_{0.08}$; f: $PBMN_{0.1}$).

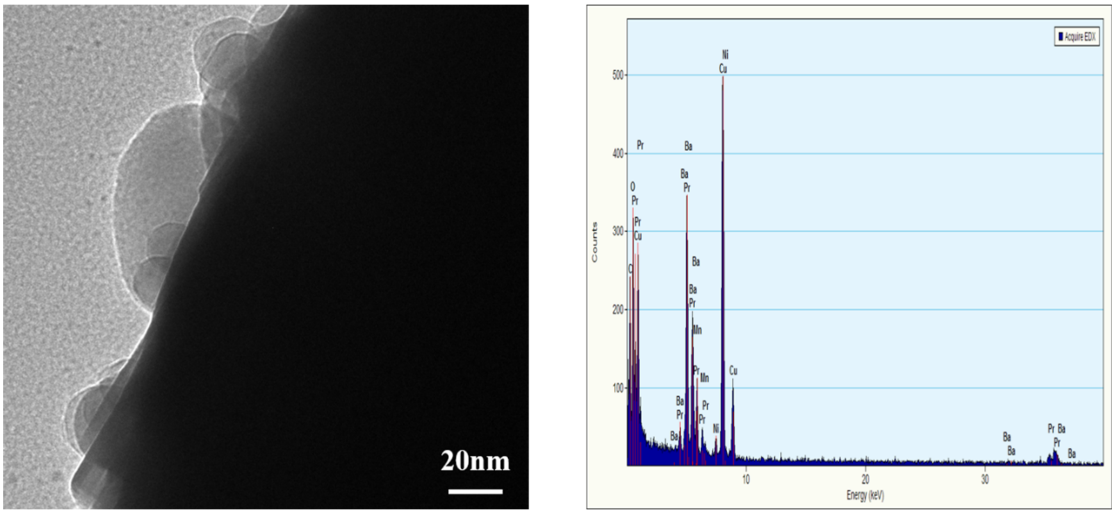

Figure S3. EDS point analysis results of reduced $PBMN_{0.1}$.

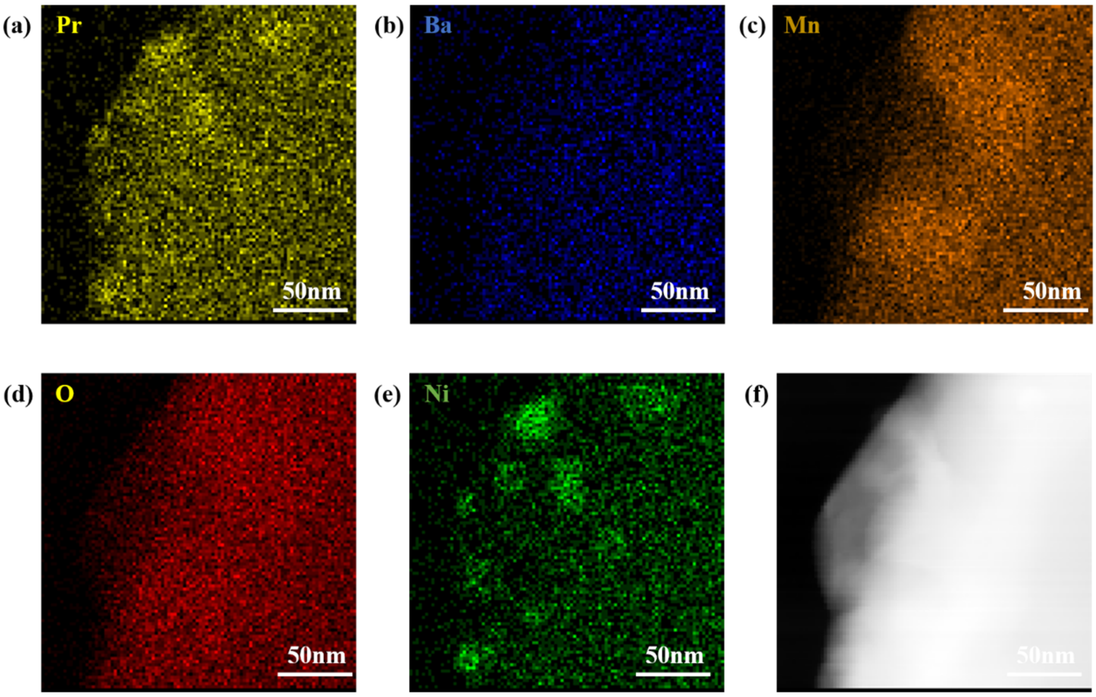

Figure S4. Mapping analysis results of reduced $PBMN_{0.1}$.

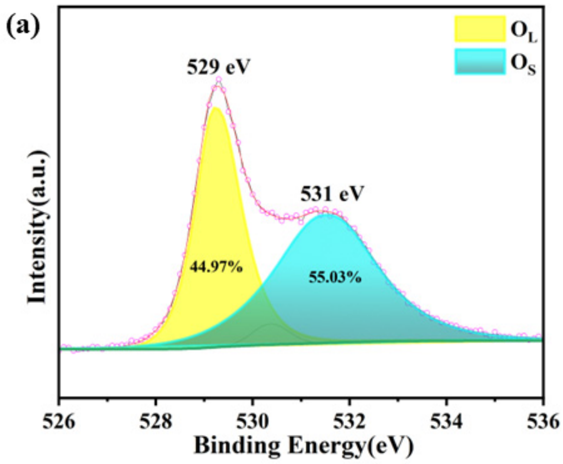

Figure S5. XPS of (a) O 1s orbitals in the oxidized state of $PBMN_{0.1}$ material; XPS of (b) O 1s orbitals in the reduced state of $PBMN_{0.1}$ material.

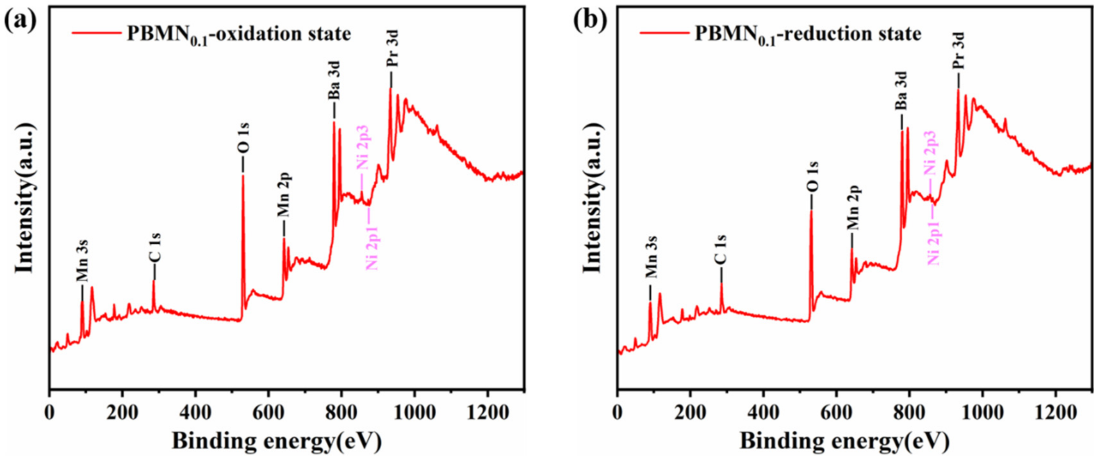

Figure S6. The XPS survey spectra of (a) $PBMN_{0.1}$ in oxidation state; (b) $PBMN_{0.1}$ in reduction state.

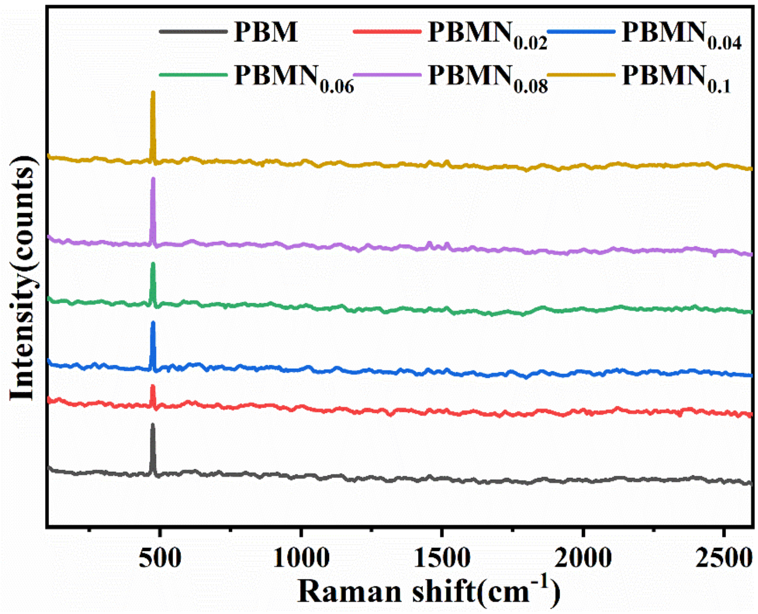

Figure S7. Raman chromatographic analysis of each component after reaction.

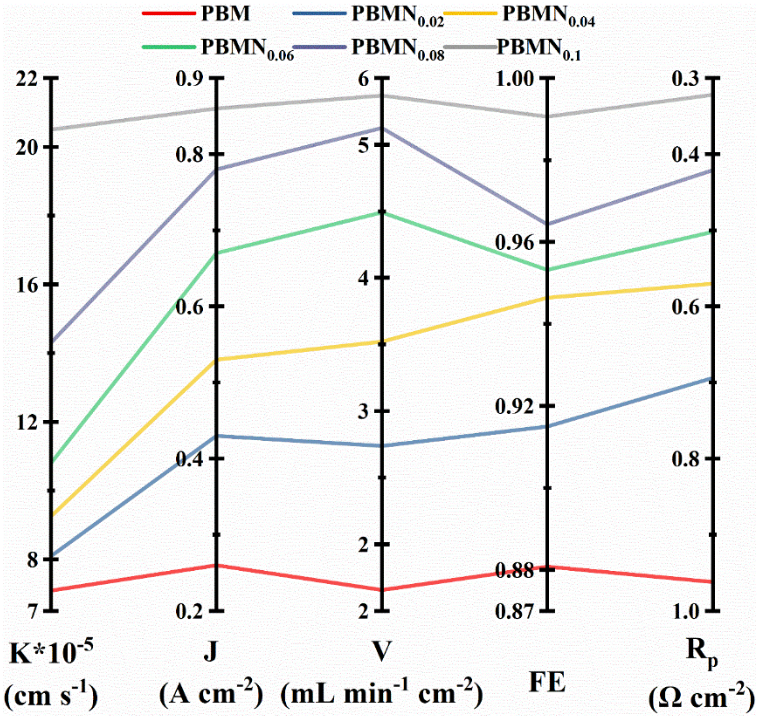

Figure S8. Performance comparison of SOEC made by different $PBMN_x$ electrode materials. K is the oxygen exchange coefficient; J is electric current density; Y is the yield of CO; FE is Faradaic efficiency; R_p is the polarization impedance.
